# The [Het-s] Prion, an Amyloid Fold as a Cell Death Activation Trigger

**DOI:** 10.1371/journal.ppat.1002687

**Published:** 2012-05-24

**Authors:** Sven J. Saupe, Asen Daskalov

**Affiliations:** IBGC, UMR 5095 CNRS Université de Bordeaux 2, Bordeaux, France; Washington University School of Medicine, United States of America

## Prions Are Amyloids

Prions are self-propagating amyloids. Amyloids are protein polymers with a cross-β structure, in which short β-strands from the monomers stack one on top of each other to make up a fibrillar polymer [1], [2]. These amyloids act as templates that convert monomers to the amyloid polymerised state. Spontaneous or chaperone-assisted fragmentation of this amyloid fibril allows multiplication of the prion particle by generating novel fibril ends where templating occurs.

Prions have initially been identified in the context of mammalian spongiform encephalopathies such as scrapie in sheep, Creutzfeld-Jacob disease in humans, or bovine spongiform encephalopathy (BSE) in cattle [3]. In these diseases, the host encoded GPI-anchored PrP prion protein turns into prion aggregates, leading to incurable lethal neurodegenerative diseases. The prion phenomenon is not restricted to this sole example. In particular, nine prion proteins have been identified in yeast and correspond to proteins with a wide range of cellular functions [4]. Generally, prion formation leads to loss of the cellular function of the protein. Yeast prions are thus detected as non-Mendelian genetic elements, leading to infectious and inheritable protein inactivation.

More than 30 human diseases, including Alzheimer disease, are caused by accumulation of amyloid aggregates of various proteins and peptides in the brain or systemic locations [2]. Many proteins can form amyloids, and the amyloid fold has been envisioned as a default conformational state that is formed when the native state of the protein cannot be efficiently attained or maintained. This view is, for instance, illustrated by the finding that inclusion bodies formed during recombinant protein over-expression in *E. coli* have an amyloid-like structure [5]. But in nature, the amyloid fold is not only found in abnormal or pathological situations. The specific physico-chemical properties of this fold have also been exploited to perform various biological functions. Amyloids constitute various cell surface structures in bacteria, fungi, and insects. Amyloids allow storage and delayed release of various peptide hormones in mammals [6]. Then, it has been proposed that this ability of a protein to exist under two states (a functional and a self-inactivating form) represents an epigenetic mechanism of gene regulation [7]. In that perspective, yeast prions have been suggested to represent a benefit [8], but this view remains debated [9].

## The [Het-s] Prion Has a Role in Non-Self Recognition

[Het-s] is a prion of the filamentous fungus *Podospora anserina* and is involved in a non-self recognition process termed heterokaryon incompatibility [10]. In filamentous fungi, cell fusions between different strains occur spontaneously and lead to the formation of heterokaryons, containing nuclei from both fusion partners. Yet, almost invariably, these heterokaryotic cells undergo cell death soon after fusion. This rejection of non-self is due to genetic differences between the fusion partners at certain specific loci designated *het* loci. The biological significance of incompatibility is not fully understood. Incompatibility could serve to limit the cytoplasmic mixing between unlike individuals to restrict transmission of mycoviruses between strains or to prevent conspeficic parasitism of one nuclear type by another. It has also been proposed that incompatibility in fungi could represent an evolutionary by-product of pathogen-driven divergence in genes whose primary function lies in host defense against microbial pathogens [11]. In this view, *het* genes were identified because they cause rejection of conspecific non-self, but their original function lies in the recognition of heterospecific non-self, a situation analogous to the MHC complex in mammals.

Nine *het* loci have been identified in *Podospora anserina* and *het-s*, the gene encoding the [Het-s] prion is one of them. The *het-s* locus has two alternative incompatible alleles, *het-s* and *het-S*. When a *het-s* strain fuses with a *het-S* strain, the fusion cell undergoes cell death. But this cell death reaction only occurs when the HET-s protein is in the prion conformation. In other words, [Het-s] prion-infected strains are incompatible with *het-S*, while prion-free strains (designated [Het-s*]) are compatible with *het-S*. The [Het-s] prion is transmitted from one strain to another after cell fusion and is transmitted from the maternal parent to the meiotic progeny [10].

## The Structure of the HET-s Prion Forming Domain Is Well Defined and Conserved in Evolution

The HET-s prion protein contains two distinct domains: an N-terminal α-helical globular domain designated HeLo and the C-terminal prion forming domain (PFD), which is necessary and sufficient for prion propagation and amyloid formation [12], [13]. This domain is natively unfolded in the soluble conformation of the protein.

The structure of the HET-s PFD in its prion conformation was solved by solid state NMR [14]. The PFD adopts a β-solenoid structure with two repeated motifs of 21 amino acids delimiting a triangular hydrophobic core. This structure is exceptionally well resolved and organised, in contrast to many other amyloid proteins, which often exist as a mixture of structural variants. A number of HET-s orthologs have been identified in other pezizomycotina fungi, and sequence comparisons and functional studies reveal a conservation of key residues, critical for formation of the β-solenoid fold, and indicate a selective pressure for the maintenance of the ability to form that specific amyloid fold [15], [16].

## HET-s Is the Trigger for Activation of the HET-S Toxicity Domain

**Figure 1:**
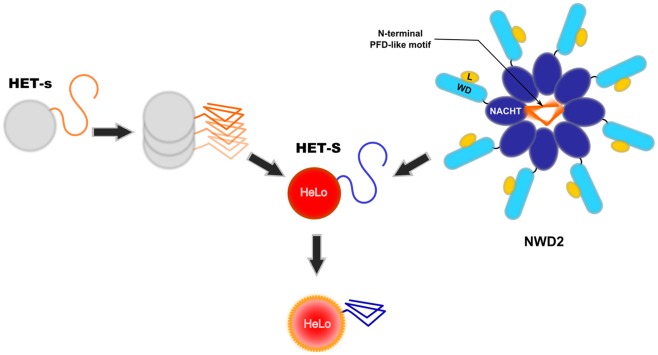
Two proposed mode of activation of the HeLo toxicity domain of HET-S. The current model envisioned for activation of the HeLo toxicity domain of HET-S is depicted. Upon prion formation, the C-terminal HET-s prion forming domain (PFD) adopts the β-solenoid amyloid fold. The β-solenoid fold of HET-s then serves as a template to transconform the corresponding region in the HET-S protein. Refolding of the HET-S PFD region leads to a refolding of the globular HeLo domain, and this refolding renders the protein toxic. The NWD2 protein displays three domains: an N-terminal motif homologous to the HET-s PFD, a central NACHT oligomerisation domain, and a C-terminal WD-repeat domain (WD). It is proposed that upon binding of a ligand (L), NWD2 undergoes oligomerisation and that this oligomerisation allows the N-terminal region of the NWD2 protein to adopt a HET-s-like β-solenoid fold. This N-terminal extension would then lead to templating and activation of the HET-S HeLo domain (in a way analogous to [Het-s]).

The HET-s and HET-S proteins differ by 13 residues, and the HET-S protein displays the same two-domain organisation as HET-s. The HET-s and HET-S PFD regions are functionally equivalent and interchangeable; the functional difference between HET-s and HET-S is determined by the amino acid differences in their HeLo domains [12]. The HeLo domain of HET-S represents the cell death execution domain in the *het-s/het-S* system. It is proposed that in the incompatibility reaction, the prion form of HET-s interacts with the PFD region of HET-S, and the conversion of the HET-S PFD region into the β-solenoid fold induces a conformational change in the HeLo domain, leading to its activation and triggering of cell death by incompatibility (Figure 1) [13]. Upon interaction with [Het-s], HET-S relocates to the cell periphery and this cell periphery localisation correlates with cell death [17]. In contrast, the HeLo domain of HET-s does not exert any toxicity. Thus, in the incompatibility reaction, HET-s and HET-S do not have equivalent roles, the HET-S HeLo domain represents the cell death execution entity, and the prion form of [Het-s] acts as a trigger for activation of this toxicity domain. Sequence analyses suggest that *het-s*-homologs in other fungal species are actually HET-S rather than HET-s homologs.

## NWD2 as a Functional Partner of HET-S

Recently, a potential additional functional partner of HET-S was identified. A search for proteins displaying homology to the HET-s PFD led to the identification of a protein termed NWD2 encoded by the gene immediately adjacent to *het-S* [18]. NWD2 is part of an NWD gene family comprising other *het* genes. NWD proteins are STAND proteins resembling Nod-like receptors and are thought to represent the fungal counterparts of pathogen recognition receptors described in plants and metazoans [19]. STAND proteins are signal transducing NTPases that undergo ligand-induced oligomerisation. They typically display three domains: a central nucleotide-binding oligomerisation domain (NOD) flanked by a C-terminal ligand-binding domain and an N-terminal effector domain. NWD2 lacks a defined effector domain and in its place displays at the N-terminal end a short region of homology with the elementary HET-s repeat motif [18]. Based on homology modelling, this region is predicted to adopt the β-solenoid fold. A model postulating the existence of a functional interaction between NWD2 and HET-S was proposed [18]. In that model, NWD2 recognises a ligand via its C-terminal WD-40 repeat domain and oligomerises in response to this binding. This oligomerisation step would put the N-terminal extensions of NWD2 proteins into close proximity and allow their cooperative folding into the β-solenoid fold. Once formed, this fold would be used as a template for transconformation of the HET-S PFD and activation of the HeLo toxicity domain. Thus, two modes of activation of the HET-S HeLo domain are now envisioned, either by interaction with the prion form of HET-s (as occurs during incompatibility) or by interaction with the oligomeric form of NWD2 (Figure 1).

This model postulates the existence of a mechanism of signal transduction STAND proteins where oligomerisation of a STAND protein induces formation of an amyloid-like fold in a short region N-terminal to the NOD domain. This amyloid-fold would in turn trigger transconformation of the protein responsible for the execution of the cell death reaction. Fungal genome analyses suggest that this mode of interaction between STAND signal transducing proteins and prion or prion-like proteins is not an isolated occurrence [18]. Several other analogous systems were identified in genome searches, suggesting that this mode of activation of effector domains is both widespread and evolutionarily conserved in fungi. What is emerging is the notion that transmission of an amyloid fold can be used as integrated part of a signal transduction pathway.

In the end, the focus needs to be redirected from [Het-s] to HET-S. [Het-s] turns out to be but a trigger to activate the HeLo toxicity domain of HET-S. HET-S is actually doing the job and represents the central player in the system. It will be of interest to elucidate the actual mechanism of toxicity that is associated with relocalisation of HET-S to the cell membrane. Other current questions regarding this system deal with the biological meaning of NWD2/HET-S pathway: what is the ligand recognised by NWD2, and what is the purpose of the HET-S-induced cell death reaction in response to this ligand binding?
